# The coronavirus pandemic: Psychosocial burden, risk-perception, and attitudes in the Austrian population and its relation to media consumption

**DOI:** 10.3389/fpubh.2022.921196

**Published:** 2022-08-10

**Authors:** Manuel Schabus, Esther-Sevil Eigl, Sebastian Stefan Widauer

**Affiliations:** Department of Psychology, University of Salzburg, Salzburg, Austria

**Keywords:** COVID-19, pandemic, mental health, fear, risk assessment, media consumption

## Abstract

**Objective:**

The aim was to assess the psychosocial burden, risk-perception and attitudes regarding the coronavirus pandemic among the Austrian population after the second infection wave in Austria.

**Methods:**

A self-designed questionnaire was available online from 17th January to 19th February 2021. Knowledge, attitudes, fears, and psychosocial burdens were collected in a comprehensive convenience sample of 3,848 adults from the Austrian general population.

**Results:**

67.2% reported their greatest fear was that a close relative could be infected; the fear of dying from COVID-19 oneself, however, was mentioned least frequently (15.2%). Isolation from family and friends (78%), homeschooling for parents (68.4%), and economic consequences (67.7%) were perceived as most stressful factors during the pandemic. Personal risk for COVID-19-associated (ICU) hospitalization was overestimated 3- to 97-fold depending on age group. Depending on the media mainly consumed, the sample could be divided into two subsamples whose estimates were remarkably opposite to each other, with regular public media users overestimating hospitalization risk substantially more.

**Conclusion:**

The results show a high degree of psychosocial burden in the Austrian population and emphasize the need for more objective risk communication in order to counteract individually perceived risk and consequently anxiety. Altogether data call for a stronger focus and immediate action for supporting mental well-being and general health in the aftermath of the coronavirus pandemic.

## Introduction

On March 11, 2020, the World Health Organization (1) announced that COVID-19 had become a global pandemic. The societal impact of this pandemic is unprecedented and affects many different areas of people's lives all over the world. The public health risks are far-reaching and do not only affect those with a severe COVID-19 course (2, 3). In addition to the health risks associated with a COVID-19 infection especially for certain groups (4, 5), large segments of the population all around the world suffered from policies designed to prevent the spread of COVID-19 (2, 6–10).

The results of an international study (98 countries) with 9,500 participants suggest that large segments of the population suffer from COVID-19-related closure (7). About 11% of respondents fell into the highest stress category, and about 50% of respondents reported only moderate levels of mental health. Social support and psychological flexibility had the greatest positive impact on respondents' well-being. However, not everyone surveyed suffered, with nearly 40% of participants reporting levels of mental health consistent with flourishing. Also quarantine measures themselves can have negative consequences for the individuals involved. A review of 24 studies showed that the majority point toward negative psychological effects such as heightened anger, confusion, or even post-traumatic stress symptoms (2). The main stressors that had a negative impact included the duration of quarantine, inadequate care, fear of infection, and the feeling of misinformation. It is meanwhile well documented that families (9), pregnant women (11), children and adolescents (10, 12, 13), as well as parents and their children with special needs (14) are affected by the negative psychosocial consequences of COVID-19 and its associated countermeasures. Children, adolescents and students, are arguably one of the most overlooked populations in the context of COVID-19. Distance learning, social deprivation, and uncertainty about consequences for their career may affect this population the most. A meta-analysis found that the prevalence of depressive symptoms (34%) and anxiety symptoms (31%) were indeed higher as compared to other groups in the population (15, 16). Particularly disadvantaged subgroups of people may suffer the most from COVID-19 and the associated changes in the living environment (8), and it has been known for long that high socioeconomic status has a positive impact on almost all health-related aspects of life (17). A study in Chile for example found that infection fatality rates were greater in low-income communities due to comorbidities and lack of access to health care (18).

Among other measures, curfews, contact restrictions (19), distance regulations, and the closure of various industries (20) and even schools (21) lead to serious and often adverse changes in the lives of many. Associated with that are fears and worries in all kinds of areas - personal, financial, economic, social and global. The effects of a life under permanent fear and uncertainty have become apparent in increased mental health issues like lower psychological well-being (22), increased rates of depression and anxiety (23) and rising numbers of insomnia symptoms (24).

Excessive levels of COVID-19-related risk perception have been shown to negatively affect individuals' mental health by increasing fear of death and decreasing happiness and positive attitudes toward oneself, life, and the future (25). Positivity, on the other hand, was positively related to happiness and negatively related to fear of death. Further results suggest that factors other than risk perception are also associated with increased fear of COVID-19 (26). Affective symptoms (which include both depressive and anxiety symptoms) and higher age also influence COVID-19 anxiety. In particular, a strong interrelation is observed between fear of COVID-19 and affective symptoms. Recently another study has shown that perceived COVID-19 anxiety is associated with increased levels of fear and greater engagement in preventive behaviors (27). An ever-increasing body of literature shows that fear and psychological distress are closely connected in COVID-19 (28, 29).

It is widely accepted that risk perception is strongly dependent on affective factors and not completely rational (30). In relation to COVID-19, indirect experiences conveyed through the media also had a significant impact on the formation of affective attitudes (31). Thus, it can be concluded that knowledge about the disease as well as the source of information can significantly influence one's individual risk perception and attitudes. In this specific context, it has already been shown that excessive media exposure is associated with greater experience of fear (32, 33) and concern (34). While the psychosocial consequences of the COVID-19 crisis are well documented in literature (2, 3, 13, 22, 24), at the time this study was planned, there were very few studies addressing knowledge and attitudes about the coronavirus pandemic in Austria. Fortunately, the situation has changed, and the Austrian Corona Panel Project (ACPP) has generated a publicly available dataset since the end of March 2020 (34). This dataset has since been collected weekly (*N* = 1,500) and is also used to study the social, political, and economic impact of the COVID-19 crisis and its associated freedom-restricting measures on the Austrian population. In order to expand the knowledge available in literature and possibly gain new insights, the present study examines the different attitudes and burdens among Austrian citizens and compares different subgroups of individuals by age and media consumption.

## Materials and methods

This study examined knowledge, attitudes, fears, and psychosocial burdens regarding the coronavirus pandemic among the general public following the second wave of infection in Austria in February 2021. The aim of the study was to obtain an overview of the psychosocial burden, risk-perception and attitudes regarding the coronavirus pandemic within the Austrian population using a comprehensive convenience sample. The survey was available online from 17 January 2021 to 19 February 2021 and the responses of 3,848 adults living in Austria were included in the analyses.

### Description of the measurements

The questionnaire consists of 38 questions, which were available *via* the questionnaire tool “LimeSurvey” (version 3.26). All users gave informed consent prior to filling out the questionnaire. The first six questions gathered demographic information about the participants (i.e., sex, marital status, employment, age group, educational qualification, diseases). After that, five questions assessed which source of information was used by participants to inform themselves about the coronavirus pandemic. Another 27 questions assessed the participants' attitudes and opinions regarding their estimation of excess mortality, perceived risk of falling ill, vaccination readiness, testing strategy, COVID-19-related measures and perceived threat, fear and resources (for more details see the original questionnaire and an English translation available at https://doi.org/10.17605/OSF.IO/T5RXB).

### Participants

Data of 3,848 adults living in Austria were analyzed (64.4% female, 35.3% male, 0.3% diverse). The main part of participants was married (44%) or in a partnership (29.2%). The remaining participants were single (20.2%), divorced (5.1%) or widowed (1.5%). Regarding the highest educational level, more than half of the sample had a university degree (45.9%) or a high school graduation (23.7%). 13.9% have done a vocational training, 11.6% had a secondary school or vocational school graduation. The remaining participants went to junior high school (3.5%), primary (0.2%) and lower secondary school (1.2%). While 54.8% reported to be employed, 14.9% were self-employed. Further 14.5% were students, 9.9% were retired, 3.3% were unemployed and 2.6% on maternity leave.

Broad advertisement of the survey in the Austrian media via the Austrian Press Agency (APA) and ORF (Austrian Broadcasting Corporation) as well as a homogeneous age distribution (see Table 1) made it possible to obtain a comprehensive overview of the current attitudes and state of mind of the Austrian society on the subject of “coronavirus.” Only the 60–69 and 70+ age groups were less represented, with 11.4%, as expected for an online survey.

**Table 1:** Age distribution in the survey and the official Austrian norm.

	**Age distribution survey**						**Statistic Austria - age distribution**					
	**Total**		**Men**		**Women**		**Total**		**Men**		**Women**	
	***n***	**%**	***n***	**%**	***n***	**%**	***n***	**%**	***n***	**%**	***n***	**%**
18–29^+^	723	18.79	243	17.91	478	19.27	1,102,195	15.28	566,215	16.12	535,980	14.49
30–39	872	22.66	306	22.55	559	22.54	1,227,485	17.02	622,254	17.72	605,231	16.36
40–49	983	25.55	316	23.29	667	26.90	1,179,382	16.36	588,713	16.76	590,669	15.96
50–59	777	20.19	282	20.78	494	19.92	1,399,348	19.40	699,717	19.93	699,631	18.91
60–69	387	10.06	158	11.64	229	9.23	1,047,888	14.53	505,874	14.40	542,014	14.65
70+	106	2.75	52	3.83	53	2.14	1,255,629	17.41	529,304	15.07	726,325	19.63
Total	3,848	100	1,357	100	2,480	100	7,211,927	100	3,512,077	100	3,699,850	100

Note. For the Austrian reference data (source: Statistic Austria), we only considered age groups above 20 years, as this corresponds to the participants of the online survey. Consequently, the six age groups shown here sum up to 100% in both parts of the table. ^+^Our data is here compared to the available 20–29 age group as officially reported by Statistic Austria for the year 2021. The official age distribution for Austria is used to weight the survey data and accounts for under-representation of people 60+.

In addition, two extreme groups of individuals were compared in the sample: those who almost exclusively consume public media (i.e., public media daily and private media at a maximum a few times per month) (*n* = 874) vs. those who in addition frequently consume private media such as “ServusTV” or “Falter” (i.e., private media several times per week and who do *not* consume public media daily, *n* = 812). In text we refer to this as public vs. private TV as this was the main source of information for the participants of the current study. With regard to public media consumption in Austria, the “Austrian Broadcasting Cooperation ORF” is the one and only public television station available and consequently the one taken into consideration by the participants of the survey. These comparisons revealed contrasting responses, which are explained below as a complement to each section. In contrast, the effects across age and gender were largely equally distributed.

## Results

### Statistical analyses

All data were analyzed with SPSS version 27 (IBM Corporation, Chicago, IL, USA). Descriptive statistics were used to examine the distribution of responses. The Chi-square test was used to evaluate statistically significant deviations from the expected distribution of responses. *Cramer's V* was provided as a measure of effect size. An alpha level of *p* < 0.05 was used for all statistical tests.

### Perceived restriction and source of media information

87.5% of the participants feel “very” (56.3%) or at least “somewhat” (31.1%) constrained by the Corona-related measures. Most participants (79.6%) share these concerns/displeasure in private with friends or family “regularly” (41.6%) or “several times” (38%). Here, all age groups are about equally critical, with the 70+ age cohort being the least concerned (73.7%) and the 40–49 age group (87.2%) being the most critical. 26.8% of participants also engage themselves publicly by posting on forums, participating in demonstrations, or even taking legal action. More than one-third of participants (37.4%) were bothered “all the time” (19.1%) or “most of the time” (18.3%) by feelings of anger and unease as they have the impression that public reports are not really objective.

Focusing on the sub-groups which differ in their media consumption, we find that 45.5% of exclusive viewers of public TV vs. 70.3% of those who also regularly consume private TV sources felt “very” constrained by Corona-related measures. A Chi-square test showed that TV consumption had a significant effect on the perceived constraint due to the COVID-19 restrictions (*X*^2^_(3)_ = 109.66, *p* < 0.001). A *Cramer's V* of 0.25^**^ confirmed this result and speaks for a moderate effect. Focusing on age, it was found that (vulnerable) groups beyond the age of 60 or 70 also feel “very” (47.6%; 70+: 39.5%) or “somewhat” (34.8%; 70+: 41.7%) restricted by Corona-measures. The results are quite similar for the younger age cohorts, with the only difference being that they seem even more restricted by the measures. Among 18- to 29-year-olds, 62.0% feel “very” or 27.0% “somewhat” restricted by Corona measures; among 30- to 39-year-olds, 61.4% feel “very” or 28% “somewhat” restricted; among 40- to 49-year-olds, 65.4% feel “very” or 26.2% “somewhat” restricted; and among 50- to 59-year-olds, 60.9% feel “very” or 28.4% “somewhat” restricted by Corona measures. Their concerns/displeasure about Corona are/is shared in private with friends or family by 65% of public TV viewers and 94.9% of private TV viewers “regularly” or “several times.” The Chi-square test showed that TV consumption had a significant effect on sharing concerns privately (*X*^2^_(3)_ = 229.80, *p* < 0.001). A *Cramer's V* of 0.36^**^ confirmed this result (moderate effect). Women and men are equally concerned and critical here, and even 72.6% of the 60 yrs group and 73.7% of the 70+ age group express their concerns about Corona-related measures and changes in existing laws “regularly” or “several times” in private.

Concerning feelings of anger and unease due to the impression that media coverage is not objective, it was found that there are significant differences between viewers of mainly public and (additionally) private television: 10.9% of public TV viewers compared with 69.2% private TV viewers rated media reports “all the time” or “most of the time” as not objective and neutral. The Chi-square test showed that the TV source had a significant effect on whether media reports are perceived as objective vs. biased (*X*^2^_(1)_ = 626.28, *p* < 0.001). A *Cramer's V* of 0.6^**^ confirmed this result and indicated a strong effect.

### Fears

The greatest fears perceived in the current pandemic are (1) that a close relative will get infected (67.2%), (2) economic damage (46.9%), and (3) the restriction of freedom of expression or of fundamental rights (46%). On the other hand, the fear of dying from the coronavirus disease was mentioned least frequently (15.2%).

Responses differed significantly in a comparative analysis of perceived fear based on what TV medium is primarily consumed to gain information about the coronavirus pandemic (cf. Table 2).

**Table 2:** Comparison of predominant fears in relation to media consumption: mainly public TV vs. also private TV consumption.

	**Public TV**	**Private TV**	***X^2^-value***	***p-value***	***Cramer-V***
Fear that a close relative will be infected	77.8%	45.6%	129.60	<0.001**	0.32**
Worries about restrictions of fundamental rights and freedom of expression	26.4%	76.0%	279.36	<0.001**	0.48**
Fear of long-term physical consequences due to COVID-19	51.5%	20.8%	108.69	<0.001**	0.30**
Fear of severe symptoms following a COVID-19 infection	55.7%	15.6%	184.48	<0.001**	0.39**
Fear of dying due to COVID-19	20.1%	8.9%	25.60	<0.001**	0.14**
Fear of psychological damage	26.2%	41.4%	29.89	<0.001**	0.16**
Fear of economic damage due to the pandemic and pandemic measures	30.6%	64.7%	132.67	<0.001**	0.33**

Note. There are significant differences between the predominant fears of the two media consumption groups. Individuals who mainly use public television report more fear regarding the health consequences of a SARS-CoV-2 infection, whereas individuals who also use private television regularly report more fear regarding psychological and economic consequences and the decay of fundamental rights. Two asterisks (**) indicate a highly significant Chi-square test result.

### Perceived burdens

The most worrisome burdens in the pandemic are: (1) not being able to maintain social contacts (77.4% not being able to meet friends, or 78.5% not being able to meet relatives), (2) home-schooling for parents (68.4%) and (3) economic consequences (67.7%). Even in the 60+ group, “not being able to meet friends or relatives in person” is ranked in the top 3 most stressful factors (80.3%). Surprisingly, the fear of being a carrier of the disease (45.1%), of falling ill oneself (24.4%) or of a lack of care due to a possible overload of the health care system (44.3%) is rated as less stressful than the previously mentioned social and economic consequences.

In the groups beyond the age of 60, not being able to meet friends (73.4%) or relatives (81.3%) in person is ranked in the top 3 most stressful factors. The other two include, just like among younger people, fear of collateral health damage (64.2%) and fear of economic harm (60.9%). Surprisingly, the fear of being a carrier of the disease (39.4%), of falling ill oneself (37.8%), or of a lack of care due to a possible overload of the health care system (45.7%) is rated as less stressful than the previously mentioned social and economic consequences.

Focusing on the two media consumption groups, we see that the four most worrisome burdens for the public TV group are (1) not being able to maintain social contacts (76.2% not being able to meet friends, or 78.5% not being able to meet relatives), (2) worries that a close relative gets SARS-CoV-2 infected (75.7%), (3) fear of being a carrier of the disease (63.7%) and (4) collateral damage for the health system such as delayed surgeries, etc. (58.6%). In contrast, the four most worrisome burdens for the private TV group are (1) economic consequences (84.6%), (2) collateral damage to the health system (79.7%), (3) not being able to maintain social contacts (79.9% not being able to meet friends, or 78.9% not being able to meet relatives), and (4) hearing/watching the news on the coronavirus pandemic (74.8%). Last but not least the subjective worry of falling ill with a SARS-CoV-2 infection varies dramatically between the two groups with 42.9% for the public TV and 7.4% for the private TV group.

### Estimated probability of falling ill

The answer to the question “How likely do you think the “Coronavirus” is to cause you a life-threatening illness (in %) over the next 12 months?” is also of interest. Based on all cases already infected with SARS-CoV-2, the statistical probability (i) of being hospitalized [official data updated from (35); Trauner and Bachner, personal communication, June 13, 2022] ranges from 1.23% (20–29 years) to 36.85% (75–79 years), and (ii) of ending up in the intensive care unit (ICU) ranges from 0.10% (20–29 years) to 5.52% (70+).

Note that the subjectively experienced risk of the coronavirus (SARS-CoV-2) causing a life-threatening illness is overestimated 3-fold (70+) to 97-fold (<29) if we equate this with ICU admission in Austria. That is, Austrian citizens aged 18–69 expect a chance of about 1:10 to encounter a life-threatening illness when they get infected by SARS-CoV-2. Scientifically, the more realistic chance of needing intensive care is at max. 1 in 230 for age 18–49, and 1:30 for an age of up to 69 according to the Austrian database (35) [updated data calculation from (35); Trauner and Bachner, personal communication, June 13, 2022; cf. Table 3]. Even lower risks are estimated by the QCovid risk calculator (UK data) from the University of Oxford (see below) (36).

**Table 3:** Risk for (ICU) hospitalization and subjectively perceived risk for a life-threatening illness due to a SARS-CoV-2 infection.

**Age**	Cases Hospital (%)*	Cases ICU (%)*	**Subjective estimate of “Corona” causing a life-threatening illness**	**Factor of subjective over-estimation of risk**	**Subjective estimate of “Corona” causing a life-threatening illness (public media subgroup)**	**Subjective estimate of “Corona” causing a life-threatening illness (private media subgroup)**
18–29^+^	1.23%	0.10%	9.65%	**97**	11.14%	7.27%
30–39	1.93%	0.22%	9.35%	**43**	13.49%	6.21%
40–49	2.95%	0.44%	8.86%	**20**	15.54%	3.95%
50–59	5.82%	1.11%	10.19%	**9**	14.63%	5.21%
60–69	15.38%	3.64%	12.78%	**4**	17.86%	6.36%
70+^+^	36.85%	5.52%	15.37%	**3**	17.73%	4.82%

* Cases hospital and Cases ICU refer to hospitalization or ICU cases (in %) of all positive (EMS)-tested cases between 01.01.2020 and 28.02.2021. Data are derived from the Austrian factsheet for COVID-19 hospitalization (35) (updated calculation Trauner and Bachner, personal communication, June 13, 2022) best matching the survey time period. +Note that some age groups from the Austrian factsheet (35) were pooled to ensure correspondence with the survey age groups. “Subjective estimate of Corona causing a life-threatening illness” is the personal estimate of risk for the total group as well as the two subgroups based on media consumption. The respective factors of risk over-estimation are printed in bold for highlighting purposes. Calculation is made as follows: Subjective estimate of “Corona” causing a life-threatening illness (%) divided by Cases ICU (%). Hot colors highlight higher values for illustration purposes.

Interestingly, however, excess mortality in the total population is not overestimated but rather underestimated. When asked about excess mortality for 2020, 27.6% assume a very high or high excess mortality but then estimate excess mortality at 3,735 cases on average (trimmed mean 3,547; 95% confidence interval 3,608–3,862). In fact, mortality was 5,350 cases higher than to be expected for 2020 in Austria. More specifically, mortality in Austria was measured at 83.386 (±2,791) cases in 2019 and at 91.527 cases in 2020. Note however, that due to fluctuations in birth rates (e.g., baby boom generation 1946–1964) and an increasing proportion of older citizens, excess mortality increases more strongly in countries with an older population - such as Austria - as compared to countries with younger citizens (37). An adjusted excess mortality rate was for example calculated for Germany, a country that is comparable to Austria in many respects (38) and estimates excess mortality for the year 2020 at about 1% across all age groups (and about 4% for 90+).

Analyzing the (i) subjective estimate of SARS-CoV-2 causing a life-threatening disease as well as (ii) the excess mortality estimates separately for the two groups primarily consuming public TV vs. those who regularly also consume private TV show vastly differing numbers. Specifically, we find higher numbers in the group of public TV viewers for (i) subjective risk with 15.16% or 11-fold overestimation (public media) vs. 5.56% or 4-fold overestimation (private media) on average (cf. Table 3) and (ii) for the proportion of people expecting very high to high excess mortality (11.14 vs. 7.27% in the youngest group [18–29 years] 17.73 vs. 4.82% in the 70+ group). This difference is statistically significant for i) subjective risk (*t*_(1576.72)_ = 14.53, *p* < 0.001, *d* = 0.67) as well as ii) excess mortality (*t* (_198.46)_ = 2.22, *p* = 0.028, *d* = 0.32) in the 18–29 up to the 70+ age cohort (*t*_(241.06)_ = 9.57, *p* < 0.001, *d* = 1.23).

### Vaccination and testing strategy

In terms of willingness to be vaccinated, 41.1% of participants said in February 2021 that they will “definitely” get vaccinated (4% of whom do so because of job requirements), 28.1% “preferred to wait” or were “still undecided,” and 26.8% responded that they will “definitely not” get vaccinated. Among those who are in favor of vaccination, 47.1% say the primary reason for vaccination is “to be able to return to a normal life.” “Protecting oneself” (31.3%) or “others” (21.6%) is less often cited as the driving factor. The majority of those who oppose vaccination (51.5%) believed that “the side effects of vaccination are not yet well enough known or researched” or “think the vaccine was approved too quickly and without sufficient studies.” 6.4% of the total sample rejected vaccination in principle. One year thereafter and according to Statistics Austria (02/22/2022), 78.2% of the whole (eligible) population had been vaccinated at least once. With regard to (valid) recovery or still valid vaccination status, the situation in Austria as of April 30, 2022, is as follows: On average, 58.2% of persons aged 18 and older are (still validly) vaccinated (but not recovered), 19% are vaccinated and recovered, another 12.3% are exclusively (valid) recovered, and 9.5% are neither vaccinated nor recovered (39). According to our data in January/February 2021, 93.1% of those who had already performed a PCR or antigen test received a negative test result. Of these, 52% had performed a PCR test and 71.3% had performed an antigen test (by February 19, 2021). Consequently, 6.9% of respondents reported having already received a positive COVID-19 test result back then. Of these positive cases, 88.2% reported having “no” or “mild symptoms,” and the remaining 11.8% reported severe symptoms without hospitalization (10.4%) or with hospitalization (1.4%).

### Return to normality

The fact that (in January/February 2021) 40.8% of the participants did not expect a return to normality until 2022 or even later can be interpreted as a lack of perspective in the general population at the time of testing. Very similar and even more alarming results are found in the infant and adolescent population (aged 6–18) in Austria (40)). The points that a majority of participants mentioned as best helping (first 2 answer ranks) through the crisis were “spending time in nature” (77.2%), “sports and exercise” (70.8%), as well as “meeting relatives or friends in person” (66.7%).

## Discussion

Altogether the results of this comprehensive online survey reflect the high degree of the psychosocial burden and anxiety regarding SARS-CoV-2 in the Austrian population. As this was an *ad-hoc* study in an online format, it can be considered a convenience sample. The subjectively estimated threat of the disease (hospitalization or mortality) is vastly overestimated and contributes to the high degree of psychosocial burdens and anxiety in the Austrian population.

In this regard, the answer to the question “How likely do you think the “coronavirus” is to cause a life-threatening illness (in %) over the next 12 months?” is of special interest. Based on all cases already infected with SARS-CoV-2, the statistical probability (i) of being hospitalized [official data updated from (35); Trauner and Bachner, personal communication, June 13, 2022] ranges from 1.23% (20–29 years) to 36.85% (75–79 years), and ii) of ending up in the intensive care unit (ICU) ranges from 0.10% (20–29 years) to 5.52% (70+) as discussed above. Yet all numbers of this kind need to be treated with caution. The most important indicator for political decisions in the COVID-19 policy in Austria and Germany were based on such data and the fear of exceeding hospitalization capacities due to COVID-19 hospitalizations. However, numerous hospitals reported all COVID-19 patients including those patients who in fact were hospitalized for other illnesses and were identified as SARS-CoV-2 infected only after already being admitted to the hospital. For example, according to Bachner et al. (35), 73% of hospitalizations in Austria have COVID-19 as the main diagnosis; even when COVID-19 secondary diagnoses that can be directly related to COVID-19 are added, this number only increases to 78–84% (35).

Similar caution may be needed when interpreting COVID-19 mortality numbers. As an example, in the German COVID-19 autopsy registry (41) 1,095 individuals who died of or with COVID-19 were analyzed. The analysis revealed COVID-19 as the underlying cause of death in 86% of the autopsy cases with 52.5% death due to COVID-19 and 33.7% death due to events subsequent to COVID-19; in 14%, patients simply had a positive SARS-CoV-2 test but it was not the underlying cause of death.

Those interested in the individual risk of severe morbidity (hospitalization) and mortality due to a SARS-CoV-2 infection in relation to individual age, sex, but also comorbidities will get accurate estimates using the University of Oxford (UK) QCOVID risk calculator (36) (based on the UK data). In order to provide an estimate of what this data looks like, a few examples are shown: 20-year-old healthy male: 1:33.333 (0.003%) for severe disease, 1:1.000.000 (0.0001%) for mortality; 30-year-old healthy female: 1:5.102 (0.021%) for severe disease, 1:200.000 (0.004%) for risk of death; 40-year-old healthy male: 1:3.300 (0.031%) for severe disease, 1:66.667 (0.002%) for risk of death; 50-year-old overweight woman (BMI 28) with type II diabetes: 1:960 (0.037%) for severe disease, 1:6.536 (0.003%) for risk of death; 60-year-old man with COPD: 1:738 (0.091%) for severe disease, 1:4.274 (0.018%) for risk of death. It is open to discussion and should be addressed in future studies why data-based risk assessments such as results from the QCovid risk calculator for the UK, RKI data for Germany, or Austrian data from the Gesundheit Österreich GmbH (35) vary so widely in these numbers. Political decisions about counter-measures to restrict the spread of the virus of course have to be based on objective data such as hospitalization and ICU admission rates, but ideally also take into account that there is a clear risk stratification for COVID-19 which is highly dependent upon age and prior comorbidities such as obesity, or anxiety and fear-related disorders (42). Yet, what appears consensual is that the risk of dying from COVID-19 is very low for individuals under 65 years of age and has been even equated to the risk of a fatal accident on the daily commute to work by car (43).

In addition, the survey revealed serious differences between those who mainly consume public service media including public TV and those who also consume private media including private TV channels. These findings extend those of Kittel et al. (34), who found people with higher exposure to public broadcasting news to be more concerned by the pandemic's developments compared to those who used this source of information less frequently. Since psychosocial effects are well known to have long-term consequences on the immune system and overall health, it is important to provide an objective and data-driven discussion of the real risks for different groups of people in the population and take countermeasures to reduce the psychosocial burden for those who are in high need of support.

Cross-sectional data from the US determined which sources of information were most trusted for health information and how reliance on specific sources was related to the adherence to recommended Corona countermeasures (47). It was found that the majority of participants relied on government sources of information such as the CDC, FDA, WHO, and local health departments. In that survey, only 36% of participants reported trusting information from social media, with white and older respondents being more likely to trust government sources.

At the peak of the pandemic, a Greek survey showed that a vast majority of respondents (93.3%) spent up to 2 h per day seeking information about COVID-19 (48). Younger respondents spent less time searching for information about the disease than older respondents. Here internet news media and television were the most common sources of information among respondents. The majority of respondents also indicated that they watch television often to very often during the day and it was seen that older people watched more television as compared to younger people, who relied more on online resources.

Another study comparing mobility data and data on trust in government at a regional level in Europe found that regions with higher trust restrict their non-essential mobility significantly more than regions with low trust (49). Alarmingly in our survey, various media consumption groups show marked differences and distrust (i) when asked about feelings of anger and unease due to the impression that media reports are not objective (11% public vs. 69% private media users) as well as in terms of (ii) individual risk assessment and burden with much stronger overestimation in mainly public media consumers (11-fold overestimation of personal risk) vs. still 4-fold overestimation in people consuming more private media.

This highlights the importance that public media and governments inform objectively and trustworthy through multiple channels in order to improve regulatory efficiency and compliance with state rules and laws. Exaggerated portrayals and biased reports, on the other hand, seem to have a significant negative impact on trust in media and politics, which, in turn, negatively influences compliance with preventive measures.

Another problem seems to be generally one of how science is communicated to the public. A study examining the extent to which liberals and conservatives are motivated to reject science that is inconsistent with their attitudes (50) found that both groups are highly motivated to interpret scientific information in a way that was consistent with their biases whereas they were more inclined to reject the scientific credibility of findings when the interpretation of the data was inconsistent with their attitudes. These results illustrate that also political attitudes can contribute to the misinterpretation or rejection of facts. In this context, it seems advisable to foster forums and platforms where open and critical discussion of all available data is possible and well communicated to the public so that well-informed and empirically data-driven opinions can form.

Tests for SARS-CoV-2 antibodies following (noticed/unnoticed) recovery were performed by only 15.9% of respondents in Austria by February 19, 2021. The latter is remarkable, as already in December 2020 SARS-Cov2 immunization and seroprevalence (measuring cumulative exposure to SARS CoV-2 infection) in the general population was estimated to be around 20% (44, 45) and after SARS-CoV-2 outbreaks like in Ischgl (Austria; March 2020) even up to 42% (46). Until February 2021, seroprevalence in the Austrian population was not systematically assessed and vaccination of the general population started in April/May 2021 independent of SARS-CoV-2 antibody status. Concerning the Austrian population's willingness to be vaccinated, a recent study with 1,350 participants reported that 70% of the 1,350 respondents thought the COVID-19 vaccine was effective in preventing and controlling the virus, with about 13% disagreeing, and 17% being unsure (data collection period: February 18, 2021 to March 17, 2021) (51). In that study, 55% were willing to adopt the vaccine when it became available, 18% did not want to be vaccinated, 17% wanted to wait, and 10% had already been vaccinated at that time. In our somewhat earlier survey comprising 3,848 Austrian adults (in January/February 2021) 44% were willing to get vaccinated, 28% were undecided or wanted to wait and 27% did not want to get vaccinated in general. According to the latest data from Statistics Austria (May 2022) (52), 50% of the population are currently vaccinated, 19% are vaccinated and recovered, 16.3% are exclusively recovered, and 14.7% are neither vaccinated nor recovered. This means that in total, 31% of the population in Austria had not been vaccinated by May 2022, which is almost identical to the number provided in our survey in January/February 2021. Note that from 5^th^ February 2022 onwards, a vaccination mandate (from the age of 18) was active in Austria; this obligation was yet suspended again on March 12^th^ and is to be re-evaluated in May 2022 (during the writing of this report).

It should be noted that this survey could unfortunately not verify representativeness in all aspects. As it is difficult to reach the elderly in online surveys, we have to mention that the 60+ groups were initially underrepresented in our data set. As it was an unfunded *ad-hoc* investigation, we unfortunately did not have the resources to reach out to elderly people directly by phone or face-to-face meetings. To adjust for this underrepresentation of people 60+ we therefore introduced a weighting factor and adjusted our outcome regarding age and gender according to the official distribution in Austria (according to Statistics Austria). Furthermore, as is true for all kind of surveys, we cannot completely rule out self-selection, or undercoverage of certain groups of the population. Yet we want to emphasize that we did all that we could in order to increase participation by broadly advertising the study in the Austrian media landscape (Austrian Press Agency, public television and newspapers, etc.). A sizeable proportion of over 3,800 people between 18 and 70+ participated in the end.

There are several other factors that could also been explored and that might limit the generalizability of the data but were not asked about in the survey (e.g., political views, migration background, social class, etc.). Critically, it should also be noted that admission to the ICU and the “subjective assessment that COVID-19 will lead to a life-threatening illness in the next 12 months” are not readily equated. The aim of this question was to obtain a subjective correlate of personal risk assessment; it is assumed that admission to the ICU is a plausible consequence of “life-threatening” illnesses. The QCovid risk assessment tool by the University of Oxford is a convenient way for any person 18+ to calculate the individual risk (including comorbidities) for hospitalization or COVID-19-associated mortality. As it is designed and validated for Great Britain, we cannot rule out that actual numbers for hospitalization and mortality might differ to some degree in Austria (according to differences in the medical system, differences in habits or overall health, etc.).

The time frame was also deliberate, as very few individuals already had COVID-19 at the start of the survey. The aim of this survey was to give as many Austrians as possible the opportunity to share their personal psychosocial burdens, concerns and attitudes related to the COVID-19 pandemic. Future research should put particular emphasis on long-lasting consequences for mental health and especially on vulnerable populations who seem to have suffered most on a psychological level.

In summarizing, we eagerly await a scientific and non-emotionalized public discussion of “lockdown” measures which had been enforced to varying degrees around the world (53). Importantly, there is a need to balance risks and potential gains for varying age groups as well as groups with or without severe comorbidity. Particularly in the groups below 65 and foremost in children and adolescents, it needs to be carefully considered whether the psychosocial burden caused by school closures, social distancing and other lockdown measures has not done more harm than good. The aftermath of the pandemic is just beginning, and the public focus should finally be turned to those indirectly harmed by the coronavirus measures in order to counteract the deterioration of well-being and mental health in the general population.

As a final note, these Austrian data (*n* = 3,848), as well as the data from Germany (*n* = 3,745) and Switzerland (*n* = 1,815), can be accessed and visualized directly at bit.ly/CovidSurvey-DACH. It can be considered a “work in progress” database, where data is made accessible to scientists and the public.

## Data availability statement

The datasets analyzed for this study can be found at https://doi.org/10.17605/OSF.IO/T5RXB.

## Ethics statement

The Corona-related surveys were approved by the Ethics Committee of the University of Salzburg (EK-GZ 122013) and conducted in accordance with the Declaration of Helsinki with healthy volunteers. The patients/participants provided their informed consent digitally to participate in this study.

## Author contributions

MS initiated the study, critically revised the article, and gave final approval for the version to be published. MS and E-SE were responsible for the conception of the article and data collection. MS, E-SE, and SW carried out the file analysis and evaluation and wrote the article. All authors contributed to the article and approved the submitted version.

## Conflict of interest

The authors declare that the research was conducted in the absence of any commercial or financial relationships that could be construed as a potential conflict of interest.

## Publisher's note

All claims expressed in this article are solely those of the authors and do not necessarily represent those of their affiliated organizations, or those of the publisher, the editors and the reviewers. Any product that may be evaluated in this article, or claim that may be made by its manufacturer, is not guaranteed or endorsed by the publisher.
